# Implementation between text and work—a qualitative study of a readmission prevention program targeting elderly patients

**DOI:** 10.1186/s13012-018-0730-0

**Published:** 2018-03-01

**Authors:** Sara Fokdal Lehn, Jette Thuesen, Gitte Bunkenborg, Ann-Dorthe Zwisler, Morten Hulvej Rod

**Affiliations:** 1 Department of Medicine, Holbæk University Hospital, Smedelundsgade 60, 4300 Holbæk, Denmark; 2 Danish Knowledge Centre for Rehabilitation and Palliative Care, University of Southern Denmark and Odense University Hospital, Vestergade 17, 5800 Nyborg, Denmark; 3 Department of Anesthesiology, Holbæk University Hospital, Smedelundsgade 60, 4300 Holbæk, Denmark; 4 National Research Centre for Disadvantaged Children and Youth, Kronprinsesse Sofies Vej 35, 2000 Frederiksberg, Denmark

**Keywords:** Implementation, Context, Prevention, Readmission, Text, Work, Institutional ethnography, Health professional

## Abstract

**Background:**

Numerous studies emphasize the importance of context in implementation. Successful implementation across the health care system depends on conditions and requirements that are often presented to health professionals through text-based materials and might present contradictory expectations to the work of health professionals. In this study, we operationalize institutional context as the text-based material, which from the perspective of health professionals, influence health care work. Via the case of a readmission prevention program for elderly patients, we examine the experiences of health professionals that work with implementation, concerning the contradictions that arise between the demands imposed by program implementation and their everyday work routines, and the role of text-based materials in these contradictions.

**Method:**

We conducted five focus group interviews among health professionals working at different locations in a single administrative region of Denmark. The 24 health professionals in our study included hospital physicians, hospital nurses, medical secretaries, municipal care managers, registered municipal nurses, and general practitioners. All focus group interviews were transcribed verbatim. Inspired by institutional ethnography, we look into text-based materials, such as written guidelines, if health professionals indicate they are important.

**Results:**

The health professionals experience that specific demands of the readmission prevention program come into conflict with the existing demands and daily work routines. *Professional resistance to control* and the *existing digital communication tools* create tensions with a program requirement for standardized enrollment of patients to the program. In addition, *the striving for autonomy* among health professionals and the high level of *mono-professional working routines* create tension with the program requirements for an additional amount of interdisciplinary work. The different demands are widely mediated by text-based materials such as the existing digital communication tools and the instructions on how to use them, and the official agreement of the role and assignment for Danish GPs.

**Conclusion:**

Successful implementation of the prevention program is affected by various tensions between the program demands and the existing health care work. Text-based materials mediate the different demands of the institutional context in to health care work and influence the process of implementation.

**Electronic supplementary material:**

The online version of this article (10.1186/s13012-018-0730-0) contains supplementary material, which is available to authorized users.

## Background

Modern healthcare is often faced with the need to alter procedures and implement new knowledge in clinical practice across health care settings, mirroring the ongoing development of treatment and care [1, 2]. The challenge of implementation has engaged researchers for decades [2, 3], and a broad range of implementation determinants have been identified as factors influencing the outcome of implementation [4]. The implementation of evidence-based practice thus faces complex organizational realities, and integration of new practices may vary substantially across organizations [5].

According to Pfadenhauer et al. [6], implementation can be understood as an actively planned and deliberately initiated effort to bring a given object (e.g., new prevention program) into action. The literature agrees that *context* has an important influence on the implementation process and outcome [6, 7]. Context can be understood as those characteristics and circumstances that surround the intervention [6]. Various authors have categorized context into areas that influence implementation processes, such as the political context, organizational characteristics, and culture [7–9]. However, context cannot be isolated from the actors who engage in it [7]. Understanding how everyday practice interacts with contextual factors in the process of implementation can help us to understand why some interventions fail to take hold in everyday practice while others are enthusiastically adopted [10]. In practice, health professionals balance the demands of externally imposed implementation processes with the routine demands of their daily work [7, 8, 10, 11]. Demands to perform, e.g., evidence-based practice and organizational efficiency, have a great influence on health care work and the everyday choices of health professionals, yet the outcomes of these processes are not always predictable [12, 13].

One way to conceptualize the dynamic relationship between context and implementation is via the sociological perspective of institutional ethnography. Thus, in accordance with institutional ethnography, we focus on *institutional context* and operationalize this as the *text-based material* that coordinates health professionals’ work [14, 15]. In institutional ethnography, an institution is defined as, “the way clusters of ruling relations interconnect around a specific function, such as health care” [16]. Text-based material mediates the institutional context; the “architecture” of institutions that for instance include the communicative infrastructures in a health care setting [17]. The investigation begins with individual experiences and uses these to explore the dynamic relationship between local work and institutional demands [18]. Institutions cut across organizational borders and link people and work together across settings [16].

Originally, institutional ethnography understands “work” as both emotional or thought work, as well as physical tasks and communicative actions in different situations of everyday life [16]. In this study, we limit our understanding of work to something acted by health professionals in their work place setting. To understand the relationship between local work processes and external influences that come with implementation programs, institutional ethnography explores the interactions between social relations and text-based materials. Health professionals receive various instructions or guidelines via text-based materials such as clinical guidelines, user manuals, or instructions for a specific intervention. Text-based material is intended to act as a coordinating pillar for the actions of health professionals, forming part of the integration of new ideas into practice [15].

Text-based materials are defined here as any type of message disseminated to a large number of people across time and space in written, recorded, or visual form [19]. However, instructions communicated by text-based materials do not always align with the demands on health professionals that arise in the actual work setting. Typically, demands from patients and management, and the experience of *disjunctures* between these different types of demands, influence the way work is actually performed. The concept of disjuncture here refers to the tensions and contradictions between different demands imposed upon health professionals in their daily work. When examining the process of implementation that does not succeed in altering the daily work routine, the experience of disjuncture between implementation and other types of demands can be a crucial factor in explaining why certain implementation processes or innovations do not take hold.

### The prevention program

In this study, a program aiming to prevent readmissions among elderly patients, which involves professionals across health care settings, is used as a case study to explore the problems of implementation. Improving the quality of care for vulnerable^1^ elderly patients has been on the political agenda of several countries for years [20]. Despite various initiatives, it has proven challenging to implement these improvements into existing health care practices [21, 22]. Here, we focus specifically on the post-discharge follow-up program, which was initiated by policy decision makers in Denmark in 2012 along with a range of other initiatives to improve quality of care for elderly patients [23]. Evaluations from various settings have highlighted the difficulties of implementing an inter-organizational health care program [21, 22], with data showing that nearly two thirds of patients did not receive the intended intervention [24]. The program entailed a high level of inter-organizational cooperation, as well as cooperation across professional groups. As such, it constitutes a suitable case to identify the interaction of various demands on the work of health professionals.

The aim of this study was to examine how health professionals perceive contradictions that arise between the demands imposed by program implementation and their everyday work routines, focusing on the role of text-based materials in mediating various types of external demands.

## Methods

The study reporting is informed by the Consolidated Criteria for Reporting Qualitative Studies (COREQ) [25].

### Setting

The Danish health care system is an open-access, tax-funded system. General practitioners (GPs) serve gatekeepers to specialized health care and are independent operators who enter into contracts with the regions [26]. Municipalities provide practical and nursing assistance to elderly peoples with functional decline. It is organized into a purchaser-provider model, where municipal care managers (purchaser) decide the form and amount of help to be provided for a person [27]. What we call here a municipality staff nurse is a visiting nurse employed by the municipality. Hospitals are responsible for most of the specialized treatment and care in Denmark and are financed through both capitation and an activity-based subsidy that is determined by the amount of patient-related activity [28].

**Fig. 1:**
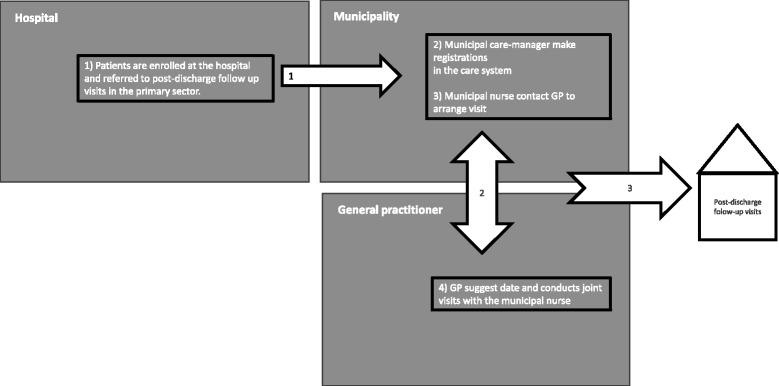
Enrollment, planning, and implementation of post-discharge follow-up visits in the post-discharge follow-up program for vulnerable elderly patients

The post-discharge follow-up program for vulnerable elderly patients to be described here has been applied to the existing health care system in Denmark. Hospitals, municipalities, and general practitioners all participate in different parts of the program (Fig. 1) (Region Zealand: Guidelines for post-discharge follow-up visits, unpublished). For each hospital and neighboring municipalities, a steering board with members from hospital, municipalities, and general practice is employed to monitor the local implementation.

Hospital medical wards screen patients who are 78 years old or older and send out a referral, usually to a municipal care manager. Municipally employed nurses coordinate joint visit with the GP with whom the patient is enrolled. The nurse and GP conduct the joint post-discharge follow-up visit in the patient’s home (Region Zealand: Agreement on post-discharge follow-up visits, unpublished). During the joint visit, the GP and municipal nurse review the patient’s hospital treatment plan, functional level, environment, and current medicine intake and then plan ongoing care.

### Data collection

Five focus group interviews were conducted in 2014, as part of a program evaluation, originally aiming at investigating “Conditions that influence the inter-organizational implementation of post-discharge follow-up visits, due to the perception of health professionals and experiences of clinical practice.” [29]. Results emerging from the first analysis were published as national report [29]. During this first analysis, it appeared that focus group participants experienced contradictions between the demands imposed by program implementation and their everyday work routines; however, it was not well covered in this first analysis. Hence, current study is a secondary analysis of the data with focus on exactly these tensions.

Focus group interviews were chosen as the data collection form in order to shed light on the social processes of implementation and to better identify the more general understandings of the program and its implementation [30]. A strategy of maximum variation sampling was used in order to obtain the experiences of the different professional groups working with the post-discharge follow-up program and to ensure geographically different settings, as well as differences in gender and level of professional experience [31]. Number of participants was pre-determined [31]. The focus group interviews were conducted in five different hospitals in one region of Denmark. Participants were located via local interdisciplinary steering boards and board members’ professional networks. Board members pointed out participants within their own organization and of the requested professions. After having been invited by their colleagues (board members), potential participants were contacted by the first author to obtain final informed consent to participate and arrange for where and when to conduct the focus group interview. These took place during the participants’ working hours. The composition of the focus groups is shown in Table 1 below. Twenty-four health professionals participated in the focus groups, yet we had seven cases of non-participation (indicated by *0* in Table 1), due to work pressure.

**Table 1:** Number of focus group participants sorted by profession and focus group

	Focus group A	Focus group B	Focus group C	Focus group D	Focus group E	Total
Hospital physician	1	0	1	1	0	3
Hospital nurse	1	3	1	1	1	7
Medical secretary	0	0	1	1	1	3
Care manager	1	0	1	1	1	4
Municipality staff nurse	1	1	1	1	0	4
General practitioner	0	1	0	1	1	3
Total	4	5	5	6	4	24

All focus groups lasted for about 2 h. They were audio recorded and then transcribed. Registered hospital nurses, hospital physicians, secretaries, municipal nurses, municipal care managers, and GPs participated in the focus groups. The first author engaged as focus group moderator, a project manager from the regional office of Quality in Care functioned as assistant moderator, and a student with qualitative training participated as an observer and took notes. The focus groups were initiated with a one-page patient story. The patient story described a fictional, yet realistic, trajectory of an elderly patient who had been admitted to the hospital and referred to a post-discharge follow-up visit (please see Additional file 1).

We included the text-based materials that were developed for the post-discharge follow-up program, e.g., guidelines and the regional inter-organizational agreement on the program, in addition to the text-based material that health professionals experienced influenced implementation (see Additional file 2).

### Analysis

As an analytical framework, we employ the concept of *sensitizing concepts*, which can draw our attention towards specific constructs in the focus group data [32]. With attention to pre-determined categories, the main author listened and read the transcribed focus group interviews several times in order to extract sections with essential meaning related to the pre-defined categories and eventually identified patterns. In this process, the main author continuously discussed the patterns and new analytical findings with the team of co-authors. Thus, inspired by institutional ethnography, we were specifically alert to disjunctures, i.e., the tensions and contradictions that arose between the professionals’ work in the triad between demands of the post-discharge follow-up program, existing work in the local setting and various text-based materials [16].

**Fig. 2:**
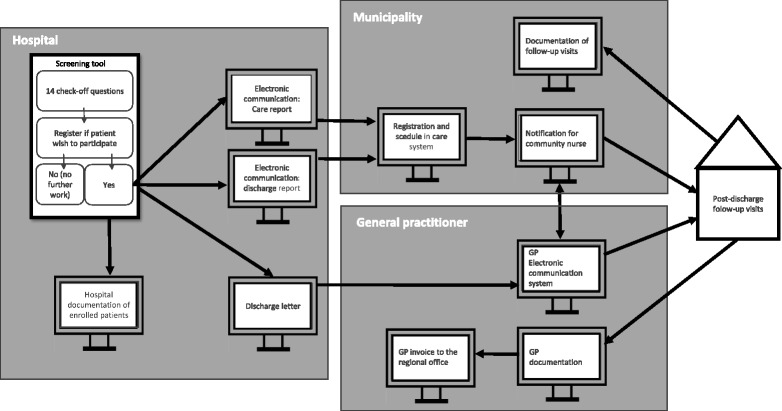
Mapping of the text-based enrollment and planning of post-discharge follow-up visits in the post-discharge follow-up program from the perspective of the health professionals

Initially, we mapped the chain of text-based communication from the enrollment of patients to the post-discharge follow-up program to a joined visit with GP and municipal nurse (Fig. 2). We did this based on both the text-based material that were developed for the post-discharge follow-up program (Additional file 2) and on information from the focus group interviews. Secondly, we went through the transcribed focus group interviews and identified the professionals’ experiences of tensions between demands of the post-discharge follow-up program and work of health professionals in general. When patterns of tensions emerged from the data, we continuously went through the transcription to identify similar experiences across focus groups, professional groups, and individuals. To clarify how tensions was influenced by other text-based materials, we included text-based material or text sequences in the analysis of tensions if *activated* by the health professionals in their descriptions of local work; where activated refers to the focus group situation, when statements mirrored knowledge from text-based materials within or outside the organization [33]. We compared the written words, with the interpretation of the focus group participants and how it affected their work.

## Results

Figure 1 shows a picture of the intended communication between hospitals, municipalities, and GPs in the post-discharge follow-up program. However, when the health professionals describe their work with the post-discharge follow-up program, a much more complicated chain of text-based communication appeared to affect professional working routines (Fig. 2). Figure 2 provides an overview and a bridge to the next level of analysis, where we unfold the health professionals’ experiences of specific work. Along with the work experiences came feelings of tension and contradiction between what was demanded as part of the post-discharge follow-up program, and the existing demands of work routines and the more general text-based materials.

**Fig. 3:**
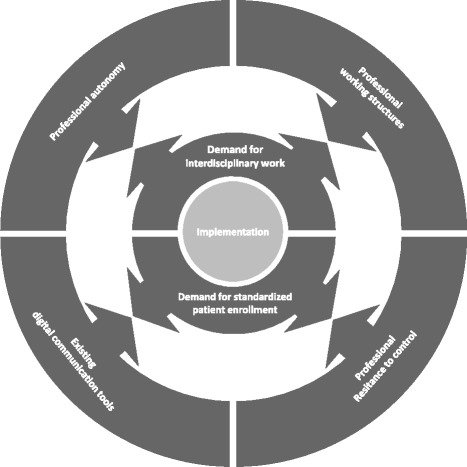
Illustration of how implementation of major demands in the post-discharge follow-up program interact with existing work routines

The next subsection below introduces the mapping of the text-based part of the post-discharge follow-up program, which is illustrated in Fig. 2. The following subsections describe what was perceived as the major demands of the program implementation and how it created tensions with existing work routines and the text-based material. Figure 3 summarize these demands and tensions. The problematized demands of the post-discharge follow-up program are illustrated in the inner circle, and the contradicting working demands are illustrated in the outer circle.

### Work with text-based material in the post-discharge follow-up program

Text-based material is the backbone of the post-discharge follow-up program. Different text need to be administered in various steps before the GP and municipality nurse can finally carry out their joint visit to the patient, and the text-based material is often supposed to initiate new work. This means that text-based material is part of health professional work, both in the form of the text-based communication they create themselves and in the form of instructions and guidelines.

The initiating screening questionnaire was paper-based (Fig. 2, top left corner), whereas the subsequent chain of text-based communication and registration was digitally based. The digital data is represented by screen vectors in the figure, and the arrows represent the professional work leading to each text-based output. The screening questionnaire required that nurses and physicians perform the screening together (see Table 2). If the screening resulted in enrollment, it needed to be registered in three different electronic databases. Hence, a code is assigned to the patient in the journal system, a note is made in the discharge letter directed to the GP, and a written referral is made in a digital communication report transmitted to the municipality at discharge. The referral addressed to the municipality is crucial, and if not sent out, the post-discharge follow-up visit will not be planned. Referral to the municipality is applied in one out of two digital communication reports that are disseminated at, respectively, admission and at discharge. The report is received by the municipality care manager, who registers the post-discharge follow-up visit in the electronic care system, which can be accessed by the municipality nurses. A municipal nurse then sends a digital request to the patient’s GP, and the GP suggests a date and time for the joint visit. When a post-discharge follow-up visit is completed, the municipal nurse documents the results in the patient’s file, and the GP, in addition to documentation, sends an invoice to the regional office. In some instances, the mapped working routines replicate the experience of conflictual demands and tensions vis a vis regular work demands of health professionals.

**Table 2:** Activated text: screening questionnaire to the post-discharge follow-up program (Region Zealand: Post-discharge follow-up screening questionnaire, unpublished)

“Instruction: If three or more check marks are obtained, an interdisciplinary decision* should determine whether the patient is to be offered post-discharge follow-up visits. The interdisciplinary decision should be made in dialogue** with the municipality whenever possible. *In the interdisciplinary decision, the following elements should be taken in to account: • Nursing needs as well as medical needs. • Magnitude of individual problems. **Applies in situations where the dialogue with municipal staff has already been obtained, i.e. planning meetings.”	

Two major demands related to post-discharge follow-up work routines emerged from the focus group interviews: *demand for standardized patient enrollment* and *demand for interdisciplinary work.* In the following subsections, we describe first the respective demand and follow up with a description of the related tensions in every day work.

### Program demand for standardized patient enrollment

As mapped in Fig. 2, hospitals alone are responsible for the enrollment of patients, a process which includes screening and referral. These requirements for patient enrollment are to be implemented in a busy environment that is separate from the local community where the post-discharge follow-up visits are conducted. The demand for new working routines creates tension with some of existing demands of professional work. The following focus group quotation came out of a discussion on whether the standardized hospital enrollment should be part of the program or not. The quote reflects the professional perception of the enrollment procedures as needlessly rigid.

GP:

“I think this project has got it wrong – I mean, we KNOW our patients. I have been a GP for many years. And I know there are also some novel GPs out there. But I know who needs this [the post-discharge follow-up program].”

From the perspective of the post-discharge follow-up program, the hospital-based and standardized enrollment ensures that enrollment terms are equal to all patients and based on the instant knowledge of the patient health and well-being. However, as the quotation above exemplifies this perspective is not shared with the main part of focus group participants and when delving into the tensions between demand for standardized patient enrollment and the daily work of the health professionals, certain issues appeared in their answers. Below, we describe two such issues: resistance to control and the existing digital communication.

#### Tension between standardized patient enrollment and resistance to control

The screening questionnaire of the post-discharge follow-up program initiates enrollment (Fig. 2). The questionnaire standardizes the process of identifying patients who would benefit from the program. Enrollment depends on fulfilling at least three criteria out of 14 screening questions, along with an interdisciplinary decision (Table 2).

The health professionals point out that the demand of the health professionals to fill in a screening questionnaire imposes limitations on their professional involvement. The standardized approach, which comes before clinical judgment (Table 2), conflicts with the professionals’ self-conception that as nurses and physicians, they are clinically competent to identify patients based on their expert judgment.

A hospital physician comments on the screening questionnaire:

“I will not sit here and fill in the front page, and then have to turn it around to look at the back page! And then, who will take over? Because apparently I can’t just hand it over to somebody else! … Well, this questionnaire is an administrative form for me! … I asked my colleagues, how often do they do it, you know. They don’t do it very often, but we have to, you know!”

The professionals emphasize awareness of how this text-based material affects their work, since the post-discharge follow-up program feeds into an existing tension between professionals’ self-conception and institutional demands to screen and register health care work. As a hospital nurse states, in a discussion on whether the screening questionnaire should be replaced by a simpler qualitative clinical judgment.

Hospital nurse:

“We are starting to realize – nobody believes in us anymore. We have to be checked in everything we do!”

The statement is delivered in an ironic tone and followed by laughter in the group, yet the nurse verbalizes a shared anxiety among health professionals that the standardization of health care work shows a lack of trust in health professionals’ skills. It elucidates how implementation is influenced by a gap between text-based instructions in the post-discharge follow-up program that create demands of standardized enrollment and the structures of professionals’ resistance towards the disempowerment of standardized working structures (Fig. 3).

#### Tension between standardized patient enrollment and existing digital communication tools

As mapped in Fig. 2, an essential step when patients have been enrolled in the post-discharge follow-up program is that patients are referred through one of the relevant digital communication reports connecting hospitals and municipalities. There is a demand for rapid standardized patient enrollment, since the work of planning and communication between the municipal health care actors is difficult and time-consuming.

The digital communication reports are standardized across hospitals and municipalities, specifying both which reports are to be transmitted and when during the hospital stay (Table 3).

**Table 3:** Guideline for the use of the “home-care and hospital communication” module, Region Zealand, November 2012 (Region Zealand: Guideline for the use of the “home-care and hospital communication” module, unpublished)

Page 4: “Generally about the module: • Care Plan (should be sent out from hospital as soon as possible after determining the treatment plan) • Discharge Report (which is sent out from hospital at time of discharge).”	

According to the Health Care Agreement [34] (text not presented here), hospitals and municipalities participate in a joint strategy to communicate digitally. It is pre-determined *when* during the hospital stay the different reports should be sent from hospital to municipality. Hospitals and municipalities have agreed to use either the *care plan* or the *discharge report* (Table 3), when referring patients to the program. The care managers and nurses highlight the importance of these forms and the timing of the forms in order to plan their work.

Community care-manager:

“They have agreed that [the referral] should be done during the Care Plan [digital communication care report].… So that we don’t risk that too much time passes before the visit is carried out. Because it was otherwise agreed that [the referral] should be part of the discharge report [digital communication discharge report]. ”

The choice of communication forms has implications for timing in both the hospital and municipality settings. Either the referral is sent out by the digital communication care plan at time of patient admission, which leaves little time to screen patients, or the referral is sent out by the digital communication discharge report at time of patient discharge, which leaves little time to coordinate home visits. Timing is additionally an important factor in the digital coordination between GPs and nurses.

A municipality staff nurse comments on the problems of interdisciplinary cooperation with GPs:

“I think that it is made difficult. Among other things because we have to use a Medcom [digital communication] system, which has a reply limit [by the GPs] of three days… And things have to go fast…”

The existing digital communication reports and the existing demands of how to manage communication tasks are experienced by staff as causing delays in the coordination of the post-discharge follow-up program. Consequently, implementation of enrollment to the post-discharge follow-up program creates tension with the strategy of digital communication (see Fig. 3).

### Program demand for interdisciplinary work

A main element of the post-discharge follow-up program is the demand for additional interdisciplinary cooperation, at both the hospital level and at the municipality level. An idea that interdisciplinary cooperation enhances quality of care is shared among professionals, in the sense that they believe that an interdisciplinary approach enhances knowledge sharing and prevents misunderstandings among health professionals, patients, and their relatives.

A hospital physician comments on the post-discharge follow-up visit:

“The interdisciplinary meeting where you, involve the patients and relatives where they are present, makes sense -- You gain from it, in my view, because the physician, when he reads the discharge letter, with his ‘medical glasses’ on, and he listens to your [nurses’] point of view.”

Nevertheless, tensions arise when the demands for interdisciplinary work are to be integrated into existing work routines. The health professionals describe two main areas of their work where the interdisciplinary cooperation approach of the post-discharge follow-up program comes into conflict with their existing work routines: the mono-professional work structures and the drive to maintain professional autonomy.

#### Tension between demand for interdisciplinary work and mono-professional working structures

The requirement for interdisciplinary cooperation as applied within the post-discharge follow-up program becomes evident for the health professionals in the initial screening stage at the hospital. The text-based material does not make it clear whether nurses or physicians are responsible for the screening, and the health professionals widely perceive the demand for *interdisciplinary judgment* (Table 2) as unclear and in opposition to the accepted organization of clinical work.

A hospital physician comments:

“It says on [the screening questionnaire] that if you manage to place three check-marks or more [the patient enrollment] has to be decided on the basis of an interdisciplinary judgement. Then I’m in trouble again… Then I have to get hold of somebody to share ‘interdisciplinarity’ with, and it’s maybe not even one of my colleagues. Because it’s somebody from the municipality. Then I’m off the train a long time ago!”

As mirrored in the quotation above, this physician resents the extra enrollment work, since the demand for interdisciplinary judgment does not mesh with the usual organization of work. Interdisciplinary meetings are indeed part of health care work, yet time is limited, and the physician has to exert extra effort to facilitate the meeting with nurse colleagues.

The hospital physician:

“Sometimes we discuss seven or eight patients at an interdisciplinary conference, … and there are six people sitting here who all want to say something… and I have to write up the minutes and I’m not so damn interested in also using so much time on this [screening]..”

Additionally, in the primary sector, the mono-professional working structures are reinforced by the fact that nurses and GPs belong to different organizations. The GPs schedule patients weeks ahead, and the municipal staff nurses are available only during the day. An example of how the health professionals perceive this logistic problem is the following exchange between a GP and municipal staff nurse:

GP: “ Well, there are many things involved in this. But, for example we have to carry out this visit within five days or something like that, right? And it [the visit] will often take place around five or six o’clock, but the nurse is not there.”

[…]

Municipal staff nurse: [interrupting] “No, we certainly don’t have the time for that during the evening shift to….”

GP “No! And often we cannot manage [to participate in post-discharge follow-up visits] …”

The National General Practice Agreement (Table 4) provides the GPs with fee-for-service, and they will profit from expanded working hours, whereas the contract for the municipal nurses is based on specific work time, where they largely do not benefit from expanded working hours. Consequently, in both the hospital and municipality settings, tensions arise between the demands for interdisciplinary work and an institutional enforcement of mono-professional working structures, which interact with the process of implementation (Fig. 3).

**Table 4:** Activated text: General Practice Agreement 2010, Denmark [40]

Chapter 1, page 12: “Roles and tasks in general practice: The basic function is to undertake independent investigation, assessment, and treatment of the major part of symptoms, diseases, and health problems, with which patients address the health care system.” §2 part 2, page 21: “Local agreements which supplement or depart from the national agreement can be concluded between a region and a general practice committee… Local agreements can count as: 1) implementation of central and local guidelines regarding role fulfillment and cooperation with the health care system in general; 2) other local agreements and/or; 3) activity agreements on new assignments in general practice.”	

#### Tension between demand for interdisciplinary work and professional strive for autonomy

Professional autonomy affects implementation of the post-discharge follow-up program differently depending on the organizational setting. Hospital physicians feel frustrated by the fact that screening is standardized and thereby controlled, and by the fact that they depend on other professional groups in the screening.

In the municipal administrative setting, the GPs’ autonomy creates tension among the staff nurses who must plan the post-discharge follow-up visits with the GP. The municipal staff nurses view the GP’s participation as depending on personal preferences, and this factor affects the nurses’ motivation.

The municipal care-manager comments on communication issues with the GPs:

“We contact them [GP’s] to say that the specific patient has been screened for [post-discharge] follow-up visits ... And then I receive a response that ‘I don’t have time’. They … always the ones who give the very brief responses…. Other [more engaged] GPs try to propose some suggestions about dates.”

GP engagement in the post-discharge follow-up program relies on the National General Practice Agreement. GPs emphasize the importance of the agreement, which defines their obligations as GPs. The General Practice Agreement for 2010–2014 describes the basic role and tasks of the GP as that of *independently* performing examination, assessment, and treatment (Table 4). However, the post-discharge follow-up program is introduced as a regional agreement, which is allowed by the National Agreement (Table 4). In the focus groups, the GPs emphasized the difference in authority between the national agreement and the local agreements.

A GP comments:

“It puzzles me that this [post-discharge follow-up] program is made operational without being a part of our [national] labor agreement. I just don’t get it! But in any case, I believe that it is very very important for general practice…… It will be so for me, it will be something I can recognize among my colleagues with the knowledge I have of them.”

As the quotation implies, this GP interprets the National General Practice agreement as the core assignment for GPs, whereas local agreements are more negotiable, with less consideration of GPs’ interests, thus resulting—in their view—in an additional work-load.

The GP continues:

“We are collapsing under the weight of projects -- all kinds! There is no limit to what kinds of projects the municipality want us to participate in. We have begun to say ‘Stop!’… We don’t even want it to be part of a paragraph two [regional agreement] and they don’t understand it at all.”

The national agreement is perceived as prioritizing the interests of the practitioners, whereas the local agreements seem to accumulate and do not pay attention to the GPs’ interests or workload. For their part, the GPs consider themselves to have a greater level of autonomy in relation to the regional agreements, and they can decline requested tasks they do not approve. Clearly, the high level of autonomy among GPs, which is supported by the General Practice agreement, has considerable influence on implementation of the post-discharge follow-up visits (Fig. 3). Hence, being dependent on the individual GP, the influence of autonomy is non-linear and might either enhance or impede implementation, depending on the individual engagement.

## Discussion

In this study, we found that the tensions arising within the daily work of the health professionals led to a situation where the work routines within the post-discharge follow-up program did not always proceed as intended. For example, not all the patients who had been enrolled by the hospital received the planned post-discharge follow-up visits from the GP and municipal staff nurse [24].

The enrollment and planning of the post-discharge follow-up program were based upon a chain of text-based communication that was supposed to mandate specific professional working routines (Fig. 2). The health professionals highlighted two program requirements that they felt created tension with their existing working conditions: the requirement for *standardized enrollment of patients*, and the *additional interdisciplinary work demands*. These requirements came into conflict with the staff’s existing work. The demand for standardized enrollment conflicted with the *general resistance to control* among health professionals and with the *existing digital communication tools*. Likewise, the program demand for additional interdisciplinary work created tension with the existing *mono-professional working structures* at both hospital and in the primary health system and with the existing effort to sustain and maintain *professional autonomy*.

The text-based materials that influenced work and thus were experienced as part of these tensions included the program materials, the digital communication tools and the instructions on how to use them, the official strategy for cooperation between the hospitals and municipalities, and the official agreement of the role and tasks for Danish GPs. According to institutional ethnography, the text-based material is something more than texts which deviate or contradict existing, accepted formulations and instructions; they mediate the institutional logic of organization of health care work [19]. For example, the digital forms and communication tools might guide how health professionals approach the patients in a hospital setting and where they focus attention [35]. Hence, text-based materials such as the screening questionnaire and the guidelines for use of digital communication mediate a wider institutional effort towards standardization and efficiency. On the other hand, health professionals experienced these institutional demands as interfering with their work environment and their professional autonomy. Professionals’ high value on autonomy is well described in the literature on professionalization [36–38]. Sena [37] elaborates how nurse autonomy is dependent on structures in the society, such as a medical dominance. Unavoidably, professional autonomy in the health field is pursued within a context of power structures among cooperating and competing professionals, where physicians generally rank higher in the professional hierarchy than nurses [37]. Our findings that hospital physicians sometimes step back from participating in the interdisciplinary work in the program seem to confirm physicians strive to retain professional power. In tandem with our findings, Sena [37] describes how professional autonomy and power structures are embedded in the institutional structures. Within the process of implementation of the post-discharge follow-up program, autonomy seemed to have a dual character. In one sense, professional autonomy enabled the health professionals to contest or even oppose the post-discharge follow-up program routines; alternatively, their autonomy allowed them to walk the extra mile to adhere to the program.

In this study, we have shown how institutional context, understood as text-based materials’ coordination of professional work, affected implementation of the post-discharge follow-up program. We found that this text-work interaction should be thoroughly considered when investigating implementation across professional settings. Further, our finding of health professionals’ striving for autonomy and their resistance to control calls for research in how health professionals can be most effectively involved in the design and implementation of health interventions [39].

It is a strength of this study that we were able to include a broad diversity of experienced health professionals and that all activated text-based materials could be identified and analyzed. However, the small number of participants (24) also imposed limits on the applicability of our study to other settings. In addition, since this study aimed to identify and describe the broad range of tensions in implementation of the post-discharge follow-up program, each of the tensions described here could be the focus of further investigation, e.g., in-depth investigation of professional autonomy and power structures at the workplaces.

## Conclusion

Within implementation research, researchers have called for studies of the dynamic role of context in implementation. In this study, we have seen how institutional context, mediated by text-based materials, take on their own meaning within the local work routine and then affect the tensions of opposing demands in implementation, enhancing or impeding it. These processes are complex, and the actual work may change from day to day. Attention towards the text-based material that by the experience of health professionals impede tensions in daily work serve as a pragmatic way to investigate the role of institutional context in implementation.

## Additional files

Additional file 1: The focus group interviews were initiated with a one page case story. The participants all read the case store and used it for a starting point for focus group discussions. (DOCX 18 kb)

Additional file 2: Different text-based materials were included in the analysis. (DOCX 16 kb)

## Abbreviation

GP: General practitioner

## Footnotes

1 By “vulnerable,” we mean those elderly patients who experience impaired functional level and who are in risk of being readmitted.
