# Supplementary material for: Implementation between text and work—a qualitative study of a readmission prevention program targeting elderly patients
Source: Implement Sci. 2018 Mar 1;13:38. doi: 10.1186/s13012-018-0730-0 (PMC5831845; doi:10.1186/s13012-018-0730-0)
Supplement: Supplementary file 1 — The focus group interviews were initiated with a one page case story. The participants all read the case store and used it for a starting point for focus group discussions. (DOCX 18 kb) [file 13012_2018_730_MOESM1_ESM.docx]

Additional file 1: Focus group interview case story

*Information for focus group participants*

*This case story is fictional. However, we intend it to be close to a real life experience. After reading this case story, we will discuss how the post-discharge follow up program proceeds in your professional work.*

**Case-story, Ingrid**

Ingrid is an eighty-two year old widow. She lives alone in an apartment for older people with disabilities. She suffers from diabetes, atrial fibrillation, and hypertension. She takes seven different medics plus vitamins on a daily basis. It can be difficult for her, to drink enough liquid. For some time, Ingrid have been feeling tired and her gait have been unstable.

**[Medication list^[[1]](#footnote-1)^]**

Ingrid receive house cleaning from the municipal home care service for one hour every second week. Additionally, she is paid a weekly visit from the registered municipal nurse to administrate medics and measure blood glucosamine level. She sees the general practitioner about once a month to control her anticoagulants and monitor her diabetes.

One day when Ingrid’s daughter is visiting, the daughter realize that Ingrid is not feeling well. Ingrid is tired and it is difficult for her to communicate. Ingrid’s daughter calls the municipal nurse, who says she will be there within an hour. When the nurse arrive, Ingrid seems perplexed and feels dizzy. Her daughter is anxious and they decide to call the ambulance.

At the hospital, it turns out that Ingrid’s pulse and blood pressure is very low. She receive liquid drop, and her heart is monitored. Blood pressure stabilize. Ingrid gets better and is discharged, with adjusted medication, after three days. The hospital refer her to the post-discharge follow-up program.

The general practitioner and municipal nurse together visit Ingrid twelve days after she had been discharged. The general practitioner was not able to find time within the intended seven days from discharge. Ingrid is happy about the visit, because she has been feeling light-headed, anxious and tired since the discharge. The general practitioner and the nurse goes through the treatment plan from the hospital, and examine her medication. The general practitioner discontinue her diuretics. They decide that the nurse should visit Ingrid again after a couple of days and report back to the general practitioner.

One week after the post-discharge follow-up visit, Ingrid is readmitted to the hospital. A care assistant found her unconscious in her home. At the hospital, they find that Ingrid’s pulse is very low. She receive liquid drop and her medication for high blood pressure is adjusted. After five days, Ingrid is discharged and it is planned for her to receive more home care.

1. Seven prescription drugs listed along with multivitamin and fish oil capsules. [↑](#footnote-ref-1)
