# Supplementary material for: Implementation between text and work—a qualitative study of a readmission prevention program targeting elderly patients
Source: Implement Sci. 2018 Mar 1;13:38. doi: 10.1186/s13012-018-0730-0 (PMC5831845; doi:10.1186/s13012-018-0730-0)
Supplement: Supplementary file 2 — Different text-based materials were included in the analysis. (DOCX 16 kb) [file 13012_2018_730_MOESM2_ESM.docx]

Additional file 2: Text-based material in the analysis

| *Table describing the text based materials employed in the analysis, structured by how they are entered into the analysis.* | |
| --- | --- |
| Text-based material | Content |
| **Text based material -- developed by the post-discharge follow-up program, and used for mapping text-based communication of the post-discharge follow-up program** |  |
| Hospital Guidelines for enrollment to post-discharge Follow-up visits in Region Zealand | Short description of what the health professionals in the hospital setting should do to coordinate post-discharge follow-up visits. |
| Inter-organizational agreement on post-discharge Follow-up visits in Region Zealand | Description of the respective responsibilities in each health care organization involved in the post-discharge follow-up program. |
| Guidelines for performance of post-discharge follow-up visit in Region Zealand | Description of the specific actions of nurses and GP’s in cooperation at the post-discharge follow-up visit. |
| **Text based material -- activated by the interview participants.** |  |
| Screening questionnaire | Screening tool with fourteen questions on patient health, treatment and social conditions, to identify patients to the post-discharge follow-up program. |
| Digital communication care plan | Standardized electronic report for the hospital nurses to communicate to the municipality: status and expectations about treatment and care, need for coordination and expected time of discharge. |
| Digital communication discharge report | Standardized electronic report for the hospital nurses to communicate to the municipality: information about admission, functional level, diagnoses, and need for continued care. |
| Guidelines for digital communication in Region Zealand | Guidelines describing how to use the digital communication tools, e.g. when during the patient trajectory to send out |
| Health Care Agreement 2010-2014 | Agreement among region and relevant municipalities: aim and vision of cooperation generally and for specific patient groups, and specific allocation of responsibilities in practice. |
| General Practice Agreement 2010-2014 | Contract between Danish Regions and the Organization of General Practice. |
